# Modulation of goat monocyte function by HCcyst-2, a secreted cystatin from *Haemonchus contortus*

**DOI:** 10.18632/oncotarget.17308

**Published:** 2017-04-21

**Authors:** Yujian Wang, Yuling Wen, Shuai Wang, Muhammad Ehsan, RuoFeng Yan, XiaoKai Song, LiXin Xu, XiangRui Li

**Affiliations:** ^1^ College of Veterinary Medicine, Nanjing Agricultural University, Nanjing 210095, PR China

**Keywords:** Haemonchus contortus, cystatin, monocyte, immunomodulation

## Abstract

Modulation and suppression of the host immune response by nematode parasites have been reported extensively and the cysteine protease inhibitor (cystatin) is identified as one of the major immunomodulator. In the present study, we cloned and produced recombinant cystatin protein from nematode parasite *Haemonchus contortus* (rHCcyst-2) and investigated its immunomodulatory effects on goat monocyte. rHCcyst-2 protein is biologically functional as shown by its ability to inhibit the protease activity of cathepsin L, cathepsin B and papain. Immunohistochemical test demonstrated that the native HCcyst-2 protein was predominantly localized at the body surface and internal surface of the worm's gut. We demonstrated that rHCcyst-2 could be distinguished by antisera from goats experimentally infected with *H. contortus* and could uptake by goat monocytes. The immunomodulatory effects of HCcyst-2 on cytokine secretion, MHC molecule expression, NO production and phagocytosis were observed by co-incubation of rHCcyst-2 with goat monocytes. The results showed that the interaction of rHCcyst-2 decreased the production of TNF-α, IL-1β and IL-12p40. However, it significantly increased the secretion of IL-10 in goat monocytes. After rHCcyst-2 exposure, the expression of MHC-II on goat monocytes was inhibited. Moreover, rHCcyst-2 could up-regulate the LPS induced NO production of goat monocytes. Phagocytotic assay by FITC-dextran internalization showed that rHCcyst-2 inhibited the phagocytosis of goat monocytes. Our findings provided potential target as immunoregulator, and will be helpful to illustrate the molecular basis of host–parasite interactions and search for new potential molecule as vaccine and drug target candidate.

## INTRODUCTION

The cystatins belongs to superfamily of enzymes which consists of evolutionary tight-binding inhibitors to form reversible complexes of papain-like cysteine proteases [1]. Based on sequence motifs and the conserved domains, cystatins are categorized into four subfamilies, the type 1 cystatins or stefins are cytoplasmic proteins lacking of signal peptides, contrary to that type 2 cystatins are secretory-type proteins, which exhibits signal peptides in its structure. Whereas, third type of cystatin proteins also called kininogens and fourth type of cystatin-like proteins named as fetuins and histidine-rich proteins [2]. It has been evaluated that proteins belong to the cystatins family are existing in a variety of organisms, including, invertebrates, vertebrates, plants and even in protozoa [3, 4]. Previous researches demonstrated that cystatins from various vertebrate species are involved in biological processes, such as immune system development, antigen presentation, epidermal homeostasis, neutrophil chemotaxis during the mechanism of inflammation and apoptosis [5–8].

The abomasal blood feeding nematode parasite *Haemonchus contortus (H. contortus)*, is an economical challenge to the livestock industry worldwide, causes severe infection that lead to anaemia, lethargy and even death of the animal, mainly in young lambs [9, 10]. The gastrointestinal nematodes or parasites which exist within tissues of their hosts continuously exposed to a range of immune effector mechanisms. To survive within the host environment, parasites has to adopt a strategy to manage within the immune responses by the release of immunomodulatory constituents that halt effector mechanisms or intermingle with the cytokine network [11]. Both complexity of mammalian innate and adaptive immune mechanisms and the long co-evolutionary association during host-parasite interface, play a considerable role in the number of molecular interactions [12]. Many studies have been conducted in recent years, which shown that cystatins from various nematode parasites act as major immune modulators during host parasite interactions [4, 13].

In the present study, a cystatin gene from *H. contortus* was cloned and a recombinant protein was produced to analyse its immune modulatory activity. We found that the recombinant cystatin from *H. contortus* (rHCcyst-2) significantly modulated goat monocyte function in multiple aspects.

## RESULTS

### Cloning and sequence analysis of HCcyst-2

**Figure 1:**
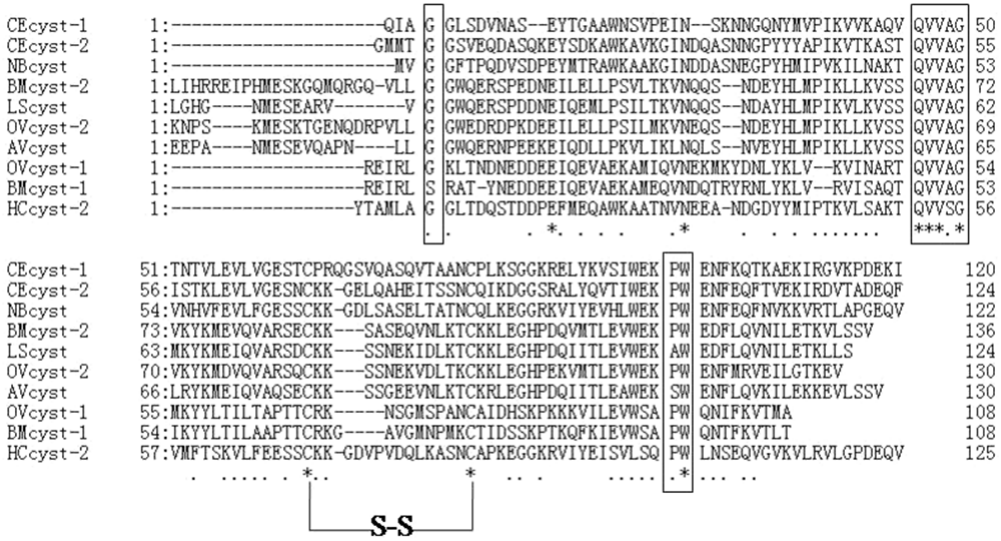
Putative amino acid alignment of HCcyst-2 with other nematode type 2 cystatins CEcyst-1 of *Caenorhabditis elegans* (GenBank accession number: AF100663); CEcyst-2 of *Caenorhabditis elegans* (GenBank accession number: AF068718); NBcyst of *Nippostrongylus brasiliensis* (GenBank accession number: AB050883); BMcyst-1 of *Brugia malayi* (GenBank accession number: U80972); BMcyst-2 of *Brugia malayi* (GenBank accession number: AF015263); LScyst of *Litomosoides sigmodontis* (GenBank accession number: AF229173); OVcyst-1 of *Onchocerca volvulus* (GenBank accession number: AF177194); OVcyst-2 of *Onchocerca volvulus* (GenBank accession number: P22085); and AVcyst of *Acanthocheilonema viteae* (GenBank accession number: L43053). The predicted signal peptide of each sequence was excluded. The conserved cystatin active sites are boxed (1: N-terminal conserved glycine; 2: QXVXG conserved motif; 3: PW conservative site).

*H.contortus* cystatin gene was cloned and designated as HCcyst-2. It contains 143 amino acid residues with an expected protein size of 14 kDa and an isoelectric point of 4.44. The sequence analysis showed that HCcyst-2 contains a conserved N-terminal glycine, QXVXG and PW motifs and one disulfide bond in its structure, which is highly conserved in most of the cystatin proteins such as cystatin 2 (Figure 1). By using SMART analysis (Schultz et al., 2000), a cystatin-like domain in the putative amino acid sequence at position 22–137 was detected. Protein sequence analysis for the predicted amino acid sequences against all non-redundant databases by using BLASTP revealed a significant similarity scores with members of the cystatin type 2 families of nematodes. The protein sequence analysis by using Signal P program detected a clear signal peptide with predicted cleavage site between amino acids 18 and 19.

### Expression and purification of rHCcyst-2

**Figure 2:**
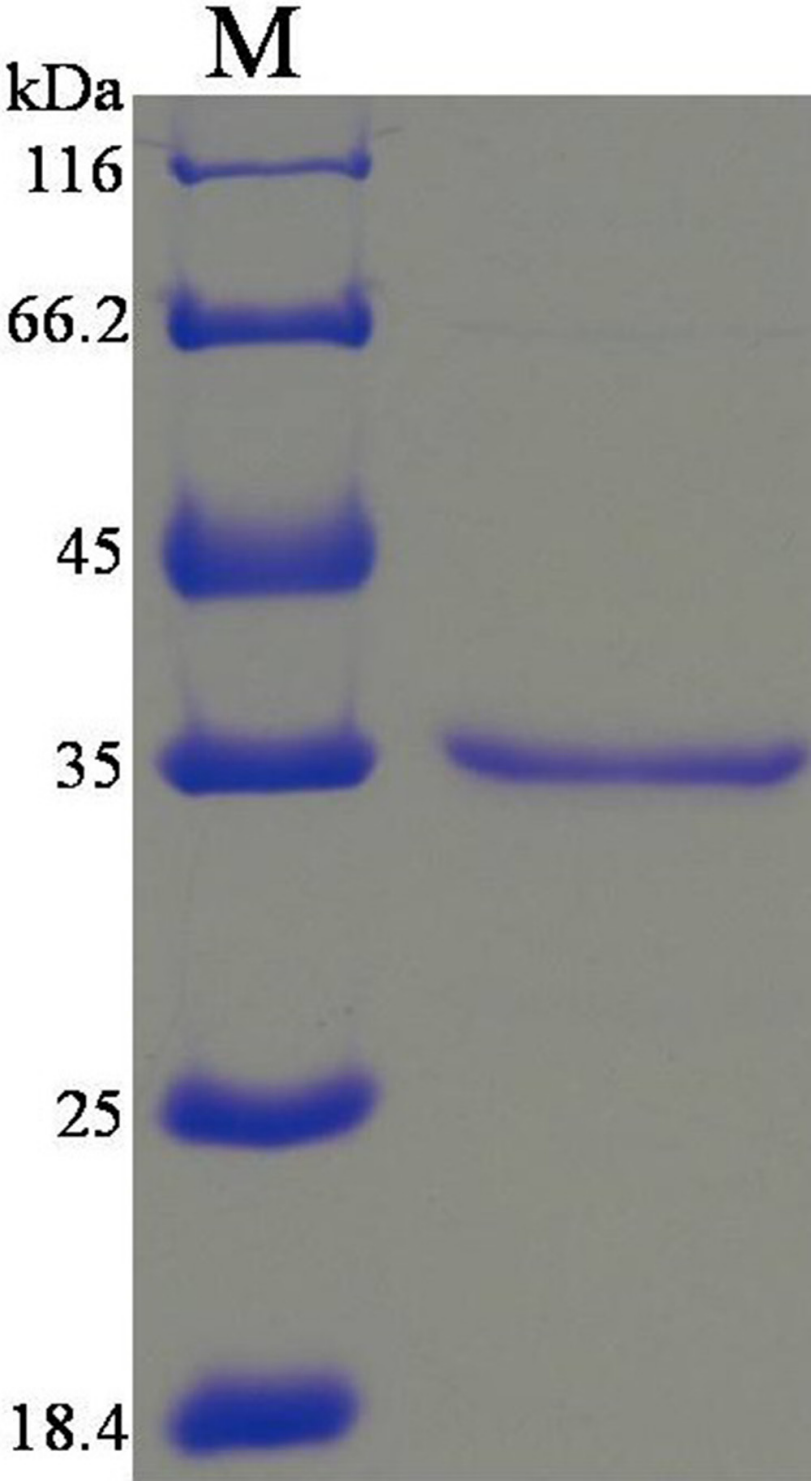

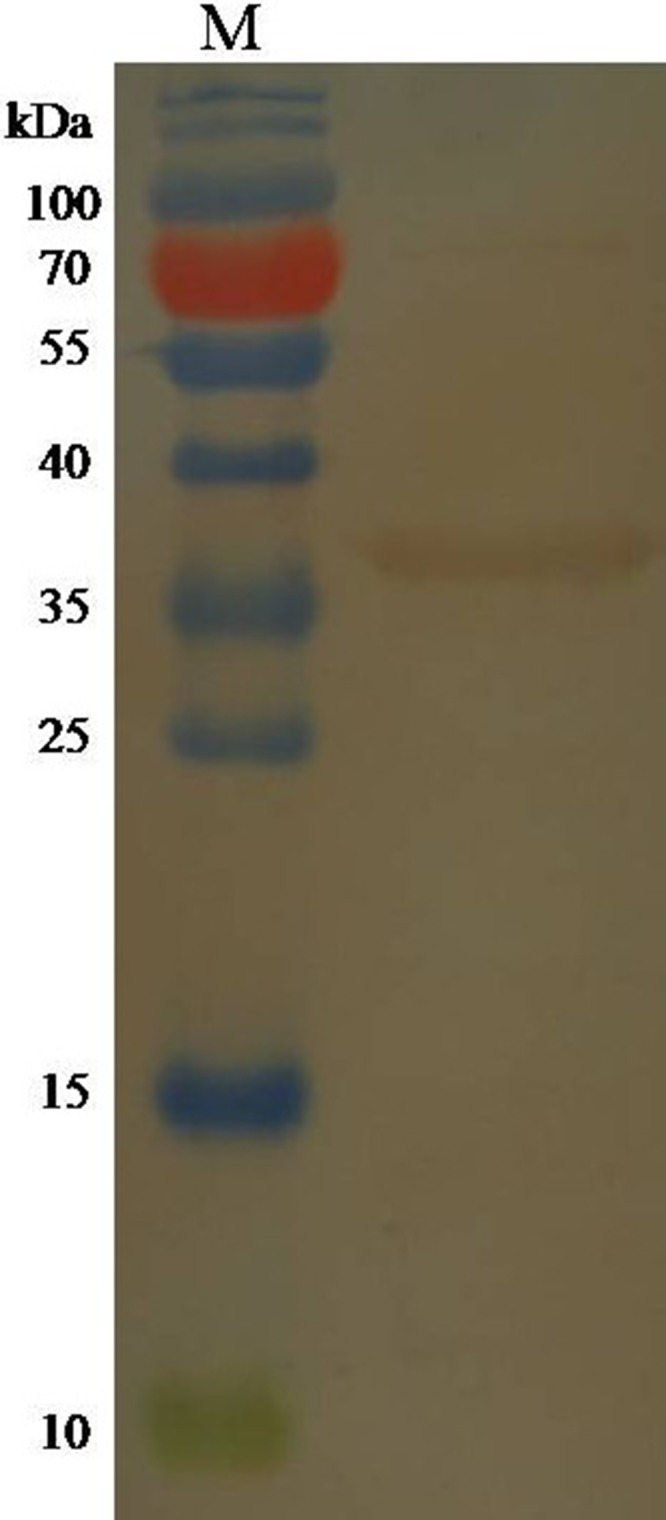
Purification of rHCcyst-2 and western blot **(A)** Purified rHCcyst-2 were resolved by SDS-PAGE on 12 % of polyacrylamide gel and stained with Coomassie brilliantblue R250. **(B)** Western blot analysis of Purified rHCcyst-2. Proteins are recognized by sera from goats experimentally infected with *H. contortus* as primary antibody.

The purified gene sequence that encodes a HCcyst-2 protein, was successfully inserted into the bacterial expression vector pET32a, and confirmed by sequence the sequence ligation into correct reading frame. The expressed recombinant protein was detected as a double His 6 tagged fusion protein with a predicted molecular mass of 34 kDa (Figure 2A). Then the rHCcyst-2 protein expressed in a soluble form was purified by affinity chromatography with His·Bind^®^128 Resin Chromatography kit (Novagen) as per manufacturer's protocol. The purified protein product of rHCcyst-2 was resolved on SDS-PAGE with estimated purity more than 95 %.

### Western blot analysis

To determine the exposure of HCcyst-2 protein to the host immune system, serum from goats experimentally infected with *H. contortus* was used as primary antibody against rHCcyst-2. Immunoblot analysis detected that rHCcyst-2 could recognized by the sera from goat experimentally infected with *H. contortus* (Figure 2B). These findings suggested that HCcyst-2 could interact with host immune system during infection process.

### Immuno-localization of HCcyst-2

**Figure 3:**
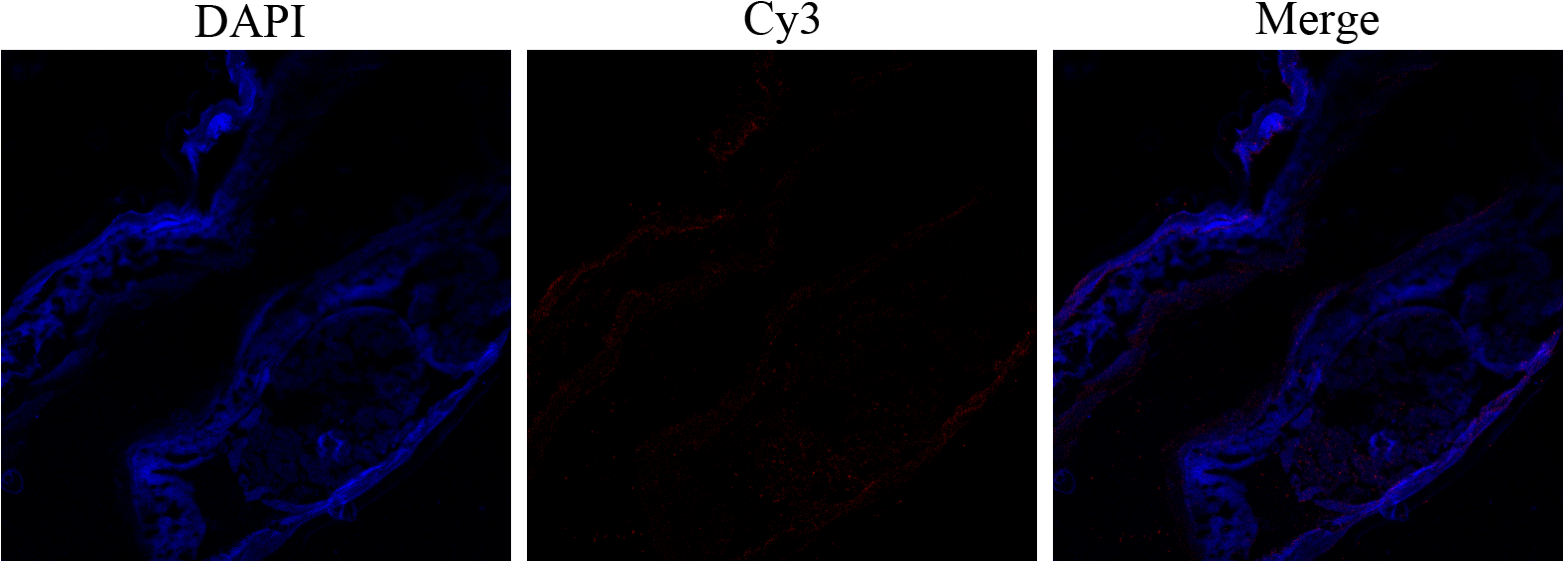
Immunohistochemical localization of HCcyst-2 protein in cryostat section of *H. contortus* HCcyst-2 protein was detected by the indirect immunofluorescence method using second antibody Cy3 labeled goat anti-rat IgG (ab6953, Abcam). The section was counterstained with DAPI to show DNA.

A partial body section of an adult female worm was used to detect HCcyst-2 protein localization through immunohistochemical analysis, as shown in Figure 3. HCcyst-2 protein and nuclei of cells fluoresced as red and blue respectively. The antibody eluted from rHCcyst-2 bound predominantly to the body surface as well as in gut surface of the worm's body (Figure 3) and no labeling was observed in control experiments.

### Proteinase inhibition assays

**Figure 4:**
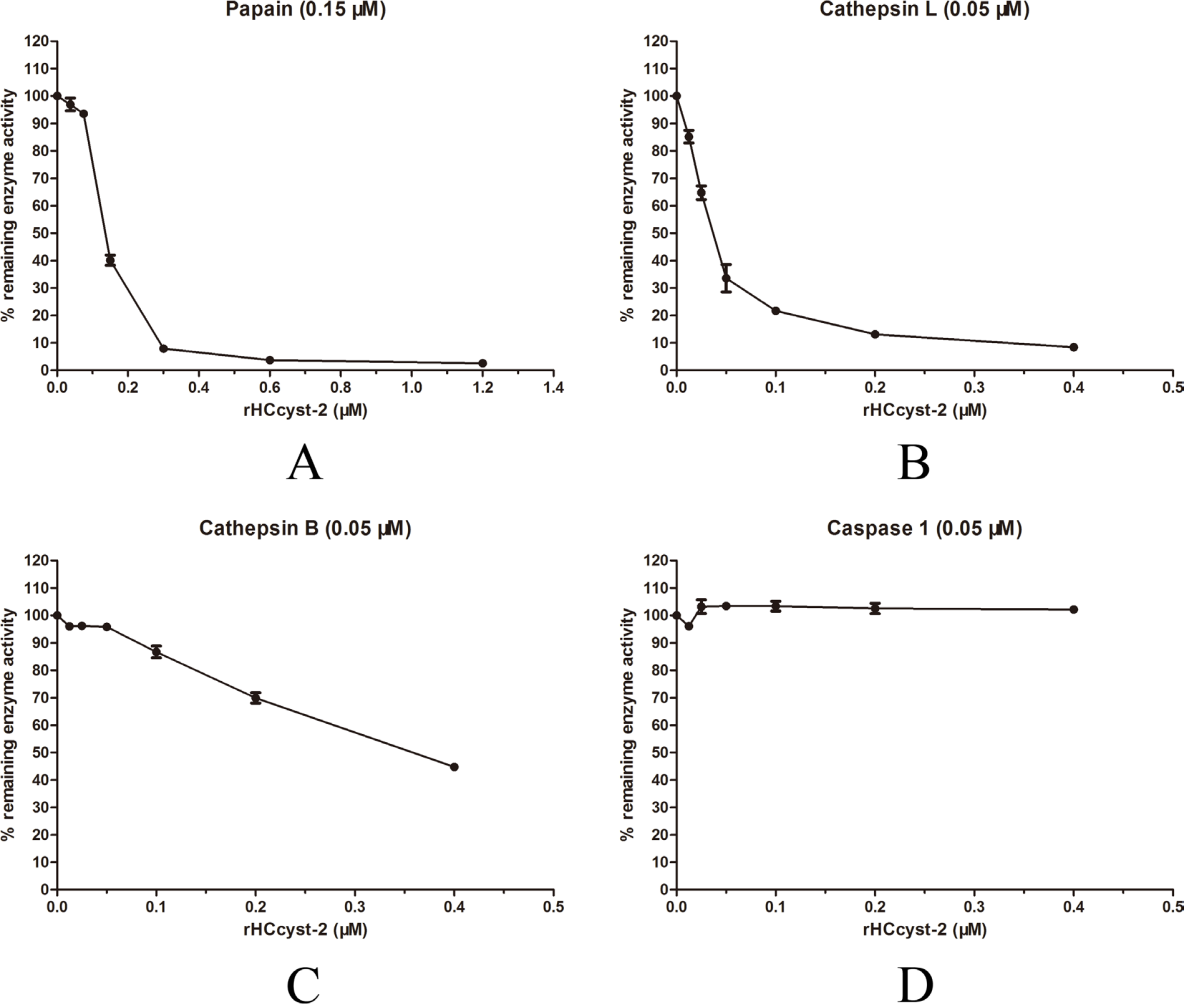
Inhibition of protease activities by the recombinant protein of HCcyst-2 **(A-D)** Cathepsin L, cathepsin B, papain and caspase 1 were incubated with each of the substrates in the presence of different concentrations of rHCcyst-2. Incubation of proteases without rHCcyst-2 resulted in 100 % enzyme activity.

The inhibitory effect of HCcyst-2 for its overlapping target enzymes was investigated by using purified recombinant cystatin against papain-like cysteine proteases and caspase 1 as another family protease to verify the target specificity of HCcyst-2. Our results highlighted that rHCcyst-2 could efficiently halt papain and cathepsin L activity, whereas rHCcyst-2 showed a lesser effective role on the inhibitory activity of cathepsin B and no inhibitory action with caspase 1 (Figure 4 and Table 1).

**Table 1:** Protease inhibition assays

Enzyme	Enzyme concentration	HCcyst-2 IC_50_ and 95% Confidence Intervals
Papain	150 nM	138 nM (136.5 to 139.5 nM)
Cathepsin L	50 nM	109.8 nM (107.9 to 111.6 nM)
Cathepsin B	50 nM	228.5 nM (215.8 to 241.9 nM)
Caspase 1	50 nM	NI

The concentration of HCcyst-2 at which 50% of the proteolytic enzymes’ activity is inhibited (IC_50_).

### Goat monocytes can uptake the rHCcyst-2

**Figure 5:**
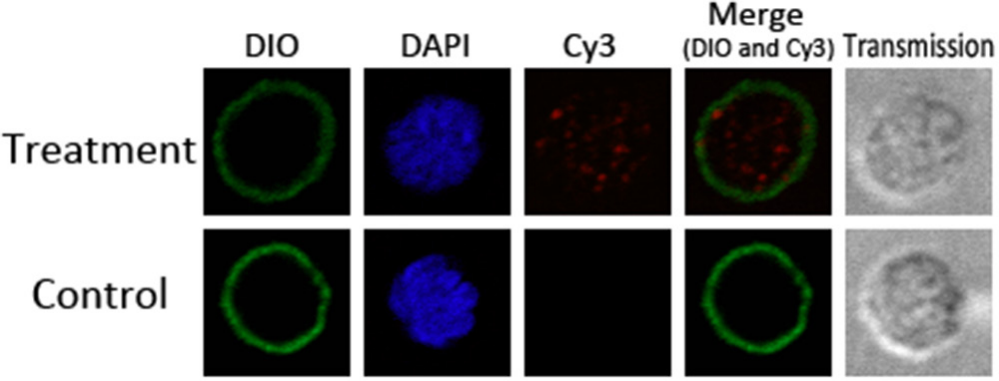
Uptake of rHCcyst-2 by goat monocytes Goat monocytes were left untreated or incubated with rHCcyst-2 (40 μg/ml) for 30 min at 37 °C. All cells were fixed and incubated with rat anti-rHCcyst-2 antibody followed by Cy3 labeled goat anti-rat IgG (red). The nuclei and membranes of the corresponding cells were visualized by DAPI (blue) and DiO (green) staining, respectively. The internalization of rHCcyst-2 by goat monocytes was visualized with a confocal laser scanning microscopy. Merge overlaps of red and green channels. The data are representative of three independent experiments.

Goat monocytes were incubated with rHCcyst-2 and the protein uptake by monocytes was investigated by an immunofluorescence approach. As depicted in Figure 5, the emission from the Cy3-labeled rHCcyst-2 was red, the DAPI-labeled nuclei were blue and DiO-labeled cell membrane was green. No fluorescence was observed under any color channel in the unstained background control. In the control group, no red fluorescence was observed (Figure 5 lower panel). Intense red fluorescence was observed when the cells were incubated with rHCcyst-2 (Figure 5 upper panel).

### The alteration of secreted cytokine levels

**Figure 6:**
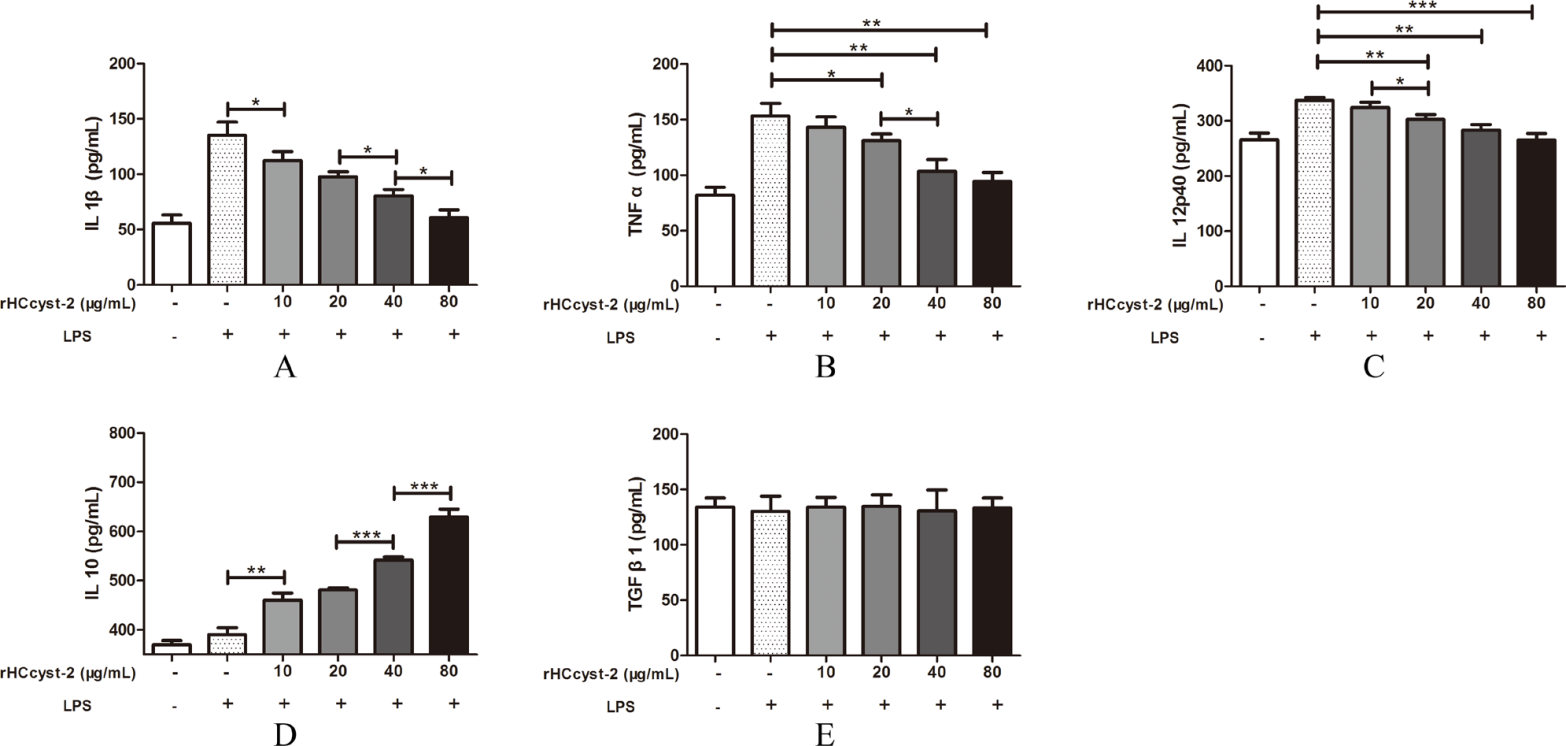
Regulation of cytokine secretion by rHCcyst-2 **(A-E)** Goat monocytes were stimulated with LPS (100 ng/ml) for 72 h in the presence or absence of rHCcyst-2. Cytokine secretion in the supernatant of cell cultures was quantified by ELISA. The data are representative of three independent experiments (*p < 0.05, **p < 0.01,***p < 0.001).

The ELISA method was used to detect the cytokines expression level in goat monocytes. Our results showed that rHCcyst-2 decreased the LPS induced production of TNF-α, IL-1β and IL-12p40 whereas, rHCcyst-2 significantly augmented the IL-10 production in goat monocytes at dose-dependent manner compared to the LPS treated only (Figure 6). No significantly difference was observed in the secretion of TGF-β1 at different groups.

### rHCcyst-2 inhibited MHC-II expression on goat monocytes

**Figure 7:**
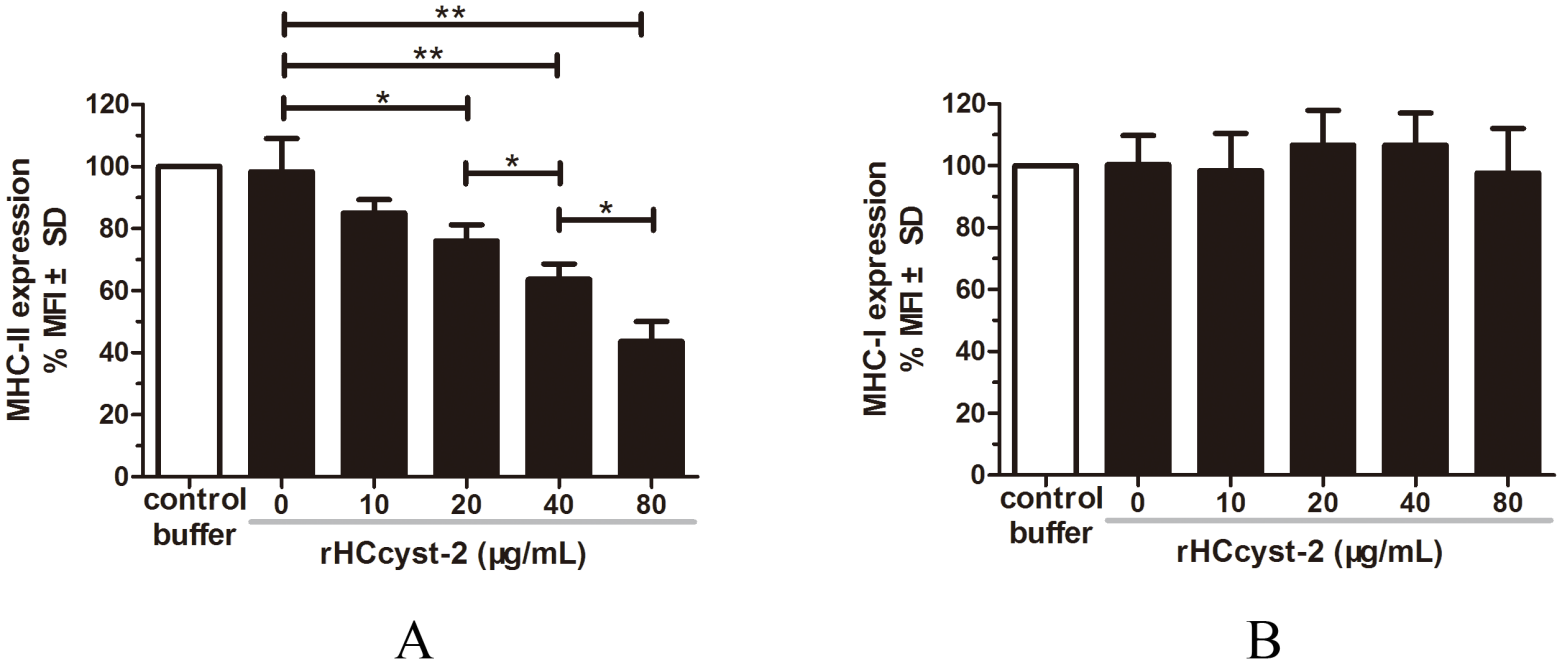
rHCcyst-2 inhibits MHC-II expression on goat monocytes Monocytes were cultured in the presence of control buffer (PBS/DTT) or different concentrations of rHCcyst-2 for 24 h. MHC-II expression was measured by flow cytometric analysis and calculated as the percentage of mean fluorescence intensity (MFI) of controls. Bars represent the MFI ± SD of controls. **(A)** MHC-II; **(B)** MHC-I. The data are representative of three independent experiments (*p < 0.05, **p < 0.01, ***p < 0.001).

MHC-I and MHC-II expressions in goat monocytes following the exposure to rHCcyst-2 were evaluated (Figure 7). The MHC-II expression in cells treated with rHCcyst-2 was significantly reduced at different protein concentrations whereas, no changes were detected in MHC-I expression in response to different concentrations of rHCcyst-2 protein, compared to the control buffer.

### NO production

**Figure 8:**
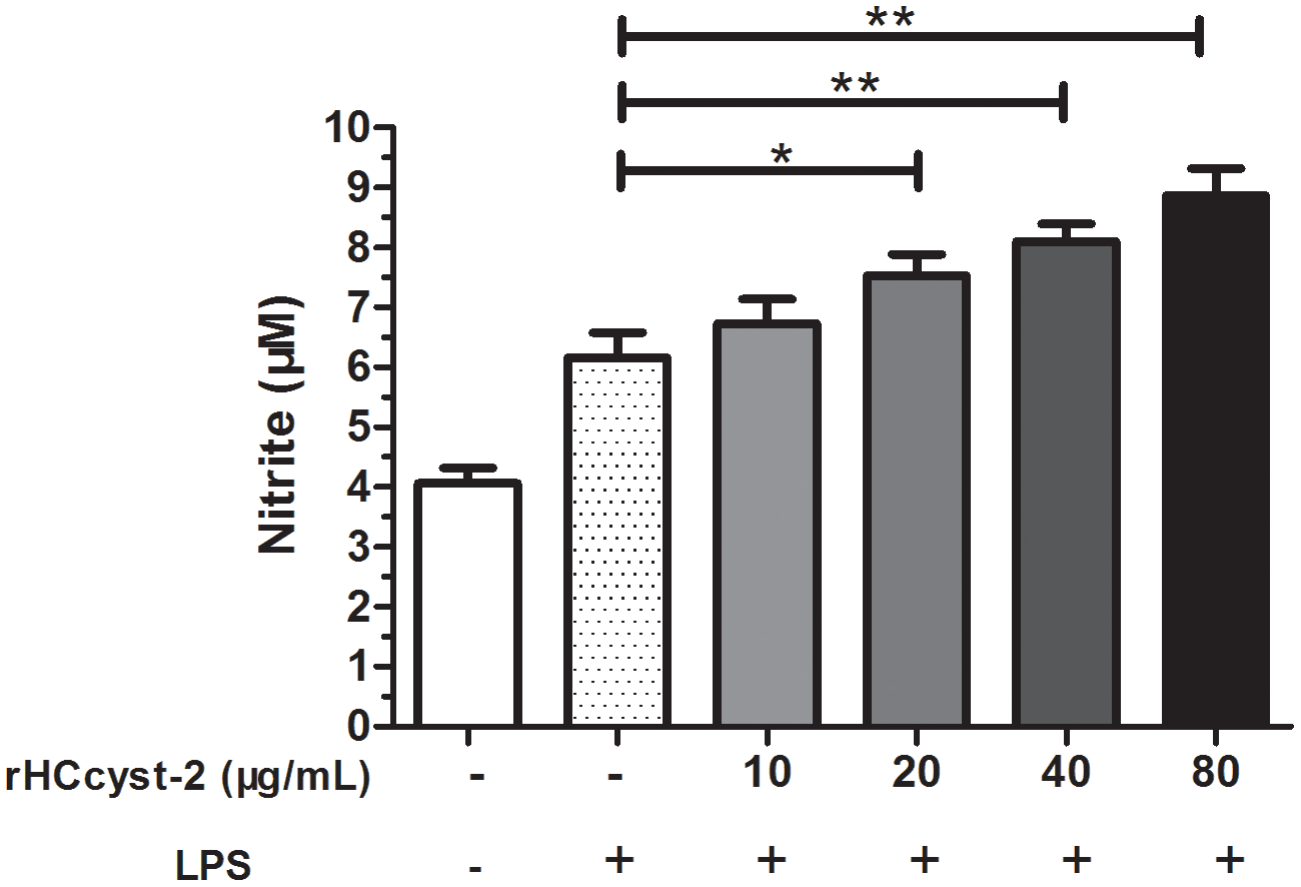
rHCcyst-2 enhanced NO production on LPS treated goat monocytes Monocytes were stimulated with LPS (100 ng/ml) for 48 h in the presence or absence of rHCcyst-2. NO was measured in the cell supernatants as nitrite using a NO assay kit. The data are representative of three independent experiments (*p < 0.05, **p < 0.01, ***p < 0.001).

The nitrate concentration of the culture supernatant was significantly increased by serial concentrations of rHCcyst-2 protein. Our results suggested that rHCcyst-2 could up-regulate the LPS induced NO production of goat monocytes (Figure 8).

### Capacity of phagocytosis

**Figure 9:**
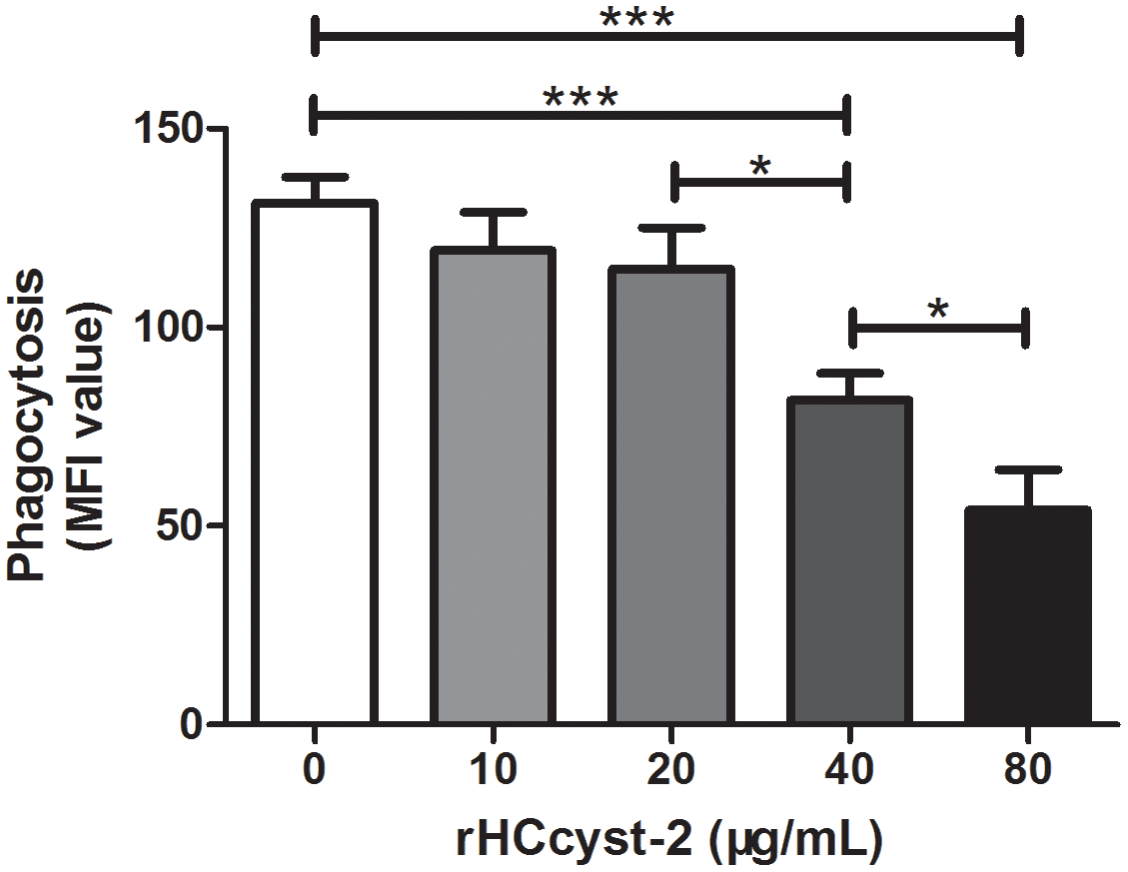
rHCcyst-2 decrease phagocytic capacity of goat monocytes Monocytes were collected after treated with rHCcyst-2 for 48 h and incubated with FITC-dextran (1 mg/ml in RPMI1640) for 1 h at 37 °C. The FITC-dextran internalization of cells was analyzed by flowcytometry and calculated as mean fluorescence intensity (MFI). The data are representative of three independent experiments (*p < 0.05, **p < 0.01, ***p < 0.001).

Phagocytic capacity of goat monocytes after 48 h treatment with different concentrations of rHCcyst-2 was examined. As shown in Figure 9, rHCcyst-2 significantly decreased the FITC-dextran uptake ability of goat monocytes in a dose-dependent manner.

## DISCUSSION

The capability of helminths parasites to modulate the immune system reinforces their stability in the mammalian host [14]. There are several reports which show the modulation of immune responses generated by the nematode parasites that inhabit in the gastrointestinal tract of their hosts [15, 16]. Cystatins associated with nematode parasites infection, have been recognized as significant immunomodulators [11]. The target cells of cystatin-induced immunomodulation seem to be monocytes, as depletion of monocytes from the PBMC reversed the inhibitory effects of *O. volvulus* cystatin [17]. In this study, we cloned a type 2 cystatin gene from *H. contortus*, HCcyst-2, produced recombinant HCcyst-2 protein and examined its immunomodulatory effects on goat monocytes.

In previous study, the characterization of various cystatins, and their capacities to inhibit the activity of cysteine proteases was evaluated. Our study showed that a histidine-tagged fused protein (rHCcyst-2) could inhibit the papain, cathepsin B and cathepsin L activity, and it also declared a strong suppressive effect against papain. The rHCcyst-2 showed comparatively lesser inhibitory role against cathepsin B, this might be due to an blocking loop that prevent the formation of substrate and inhibitor complex at the active site of cathepsin B [18]. Previously, many studies showed the absence of inhibitory action for number of cystatins [15, 17, 19].

As compared to type 1 cystatins, the type 2 cystatins are secretion-type proteins that possess signal peptides in its structure. Previously, Type 2 cystatins were demonstrated as excretory/secretory (ES) products of parasites from *Nippostrongylus brasiliensis*, *Heligmosomoides polygyrus*, *Litomosoides sigmodontis* and *Brugia malayi* [12, 15, 20, 21]. In present study, we found that rHCcyst-2 could be detected by the sera of goats experimentally diseased with *H. contortus* and the native HCcyst-2 protein was localized externally as well as internal surface of the parasite's gut. Helminths parasite cystatins play immune modulating functions based on a hypothesis that host monocyte/macrophage could uptake parasite cystatins [22]. Further, we demonstrated that goat monocyte could uptake rHCcyst-2 *in vitro*. All of these results indicated that *H. contortus* type 2 cystatins are excretory/secretory antigens capable to intermingle with the host immune mechanisms during host-parasite interactions. The continuous secretions and desired concentrations of these ES molecules are suggested to be a basic requirement for immunomodulatory functions. However, how the HCcyst-2 accrues to the functional concentration *in vivo*, and the real mechanisms or pathways involved in host parasite interactions during natural infection of *H. contortus* are worthy for further studied. Besides of their inhibitory activity on proteases, the nematode cystatins also exert substantial effect on the host's cytokines production. Previously, it was investigated that filarial cystatins play a key role to induce several cytokines expression levels which could lead anti-inflammatory responses [11]. A previous investigation showed an increased level of TNF-α response and IL-10 production in human PBMCs whereas, a decreased level of the IL-12 cytokine was found in response to the *O. volvulus* cystatin [17]. The antigen-specific expression of IFN-γ and IL-4 was decreased in mice splenocytes treated with rNbCys compared to mice treated with control protein. The mice bone-marrow-derived dendritic cell stimulated with Toll-like receptor ligand CpG, showed decrease level of TNF-α and IL-6 cytokine expression in presence of rHp-CPI. Our results showed that rHCcyst-2 decreased the LPS, induced production of TNF-α, IL-1β and IL-12p40 in goat monocytes. However, rHCcyst-2 significantly induced the production of IL-10 in goat monocytes in a dose-dependent manner compared with LPS treated only. The cytokines profile modulated by rHCcyst-2 contributes to induce an anti-inflammatory environment which is favorable for the survival of worms.

NO being a non-cytokine based immune cell-derived molecule has been suggested to involve in majority of immune responses during parasitic infections [23, 24]. Another important biological function of the NO was demonstrated to regulate the innate immune response against parasite protozoa in context of retard growth and inhibition of invasion process into the host [25–28]. Apart from its diverse biological functions NO has been suggested as an inhibitor for lymphocyte proliferation *in vitro* and a key modulator for the regulation of cytokines expression in various cell types [29, 30]. In the present study, rHCcyst-2 significantly enhanced the NO production by LPS treated goat monocytes. Previous studies showed that the NO production in IFN-γ-activated murine macrophages was substantially induced in various members of the cystatin superfamily such as human stefin B, rat T-kininogen and chicken cystatin [24]. This possibility has not been shown for synthetic inhibitors like E64; a natural cysteine protease inhibitors, to increase the NO production [31]. Interestingly, cystatins superfamily of nematodes compared to other members of the cystatin, potentially up-regulate the NO production in IFN-γ activated macrophages regardless the parasitic or free-living state of parasites [4, 31, 32]. In previous study, the decreased level of antigen-specific T cell proliferation in a murine model of filariasis was associated with NO production [33]. In several previous studies, it was revealed that parasitic nematode cystatins can inhibit antigen-specific T cell proliferation *in vitro* and *in vivo* [20, 32, 34]. The up-regulation of NO production in LPS treated goat monocytes in the presence of rHCcyst-2 might suggest a T cell proliferation inhibition event, occurs when HCcyst-2 released by parasites in parasitic stage. However, we guess further study be needed.

The phagocytosis mechanism of the immune system, largely depends upon an effective role of phagocytic cells in the elimination of pathogen/disease causing agents [35]. In this context the macrophages play an important role because of their ability to engulf and kill pathogens to cure disease [36]. In the present study, phagocytic capacity of goat monocytes was decreased after treatment with different concentrations of rHCcyst-2 in a dose-dependent manner.

The cysteine proteases of lysosomes and endosomes of antigen-presenting cells have been identified to implicate in the maturation of MHC-II molecule as well as progression of protein antigens. It has been suggested that cathepsin S plays an vital role in the series wise break down of invariant chain of proteolytic enzymes, that protects the MHC-II molecule from premature binding of antigen peptide and also involved in regulation of intracellular trafficking of MHC-II molecule [37]. Meanwhile, cathepsin B and C are essential to promote antigen peptides binding to the MHC-II peptide binding groove [38]. A previous study reported that, cystatin protein from *Nippostrongylus brasiliensis* in splenocytes of mice had a negative effect on ovalbumin protein processing by lysosomal cysteine proteases [15]. Additionally, the presence of cystatin of *O. volvulus* caused a significant reduction of human leucocyte antigen (HLA-DR) on human monocytes by 72 % to that of an *O. volvulus* control protein. In the present study, we demonstrated that rHCcyst-2 decreased the level of MHC-II expression on goat monocytes. However, rHCcyst-2 showed no change in expression of MHC-I molecule in a dose dependent manner.

In conclusion, our results showed that rHCcyst-2 could uptake by goat monocytes and exerts its immunomodulatory effects on multiple aspects to facilitate the immune evasion of *H. contortus*. These findings provided insight into the interactive relationship between parasitic nematode cystatins and host monocytes. It also shed new light on the molecular mechanisms of helminthic immune evasion. These findings may represent potential target as immunoregulator, and will be helpful to illustrate the molecular basis of host–parasite interactions and search for new potential molecule as vaccine and drug target candidate.

## MATERIALS AND METHODS

### Ethics statement

The experiment was conducted following the guidelines of the Animal Ethics Committee, Nanjing Agricultural University, China. All experimental protocols were approved by the Science and Technology Agency of Jiangsu Province. The approval ID is SYXK (SU) 2010-0005.

### Parasites and animals

*H. contortus* strain (designated Nanjing 2005) was originally obtained from Nanjing (Jiangsu Province, China) and maintained by serial passage in 3-6 months old helminths-free goats [39]. Third stage larvae (L3) used for challenge were cultured from the feces of the mono-specifically infected goats at 26 °C and stored in water at a concentration of 2500 larvae/ml at 4 °C.

Local crossbred male goats (3-6 months old) from the teaching and research flock at Nanjing Agricultural University were housed indoors in pens containing six goats per pen. The male goats were fed hay and whole shelled corn and provided with water ad libitum. All goats were dewormed twice at 2 week intervals with levamisole (8 mg/kg bodyweight) orally at the time of housing to remove naturally acquired strongyloides infection. After 2 weeks, fecal samples from each goat were examined by microscope for helminths eggs, according to standard parasitological techniques. Goats exhibiting no eggs were used in the subsequent study and daily health observations were performed throughout the experiment.

SD rats (body weight~150 g) were purchased from Experimental Animal Center of Jiangsu, PR China (Qualified Certificate:SCXK 2008-0004) and were raised in a sterilized room and fed sterilized food and water.

### Cloning of HCcyst-2 and bioinformatics analyses

Utilizing resources from online database, the open reading frame (ORF) of cystatin-like gene (GenBank accession number:CDJ82248.1) without signal peptide sequence was amplified by reverse transcription-polymerase chain reaction (RT-PCR) using designed specific pair of primers (forward:5′-TA*GAATTC*TACACAGCAATGCTGGCTG-3′ and reverse: 5′-TA*CTCGAG*GACCTGCTCATCAGGACCA-3′), in which the *EcoR*I and *Xho*I restriction sites respectively, were introduced and are shown in italics here. Following ligation of the obtained RT-PCR product with the pMD19-T vector (Takara, Dalian, China) to form pMD-cystatin, the cystatin fragment was cleaved from pMD-cystatin by *EcoR*I and *Xho*I and subcloned into the corresponding sites of pET32a vector (Invitrogen, Carlsbad, CA, USA). The accuracy of the insertion in the resulting plasmid was confirmed by sequencing.

### Expression and purification of rHCcyst-2 in *Escherichia coli*

The expression of the recombinant fusion protein in *E. coli* BL-21 cells (DE3) was induced by Isopropyl-β-D-thiogalactopyranoside (IPTG) at a final concentration of 1 mM for 6 h at 37 °C in Luria-Bertini (LB) medium with ampicillin (100 μg/ml). The histidine-tagged fusion protein was purified from the supernatant of bacterial lysates using the His·Bind^®^128 Resin Chromatography kit (Novagen) according to the manufacturer's instructions, and dialyzed in phosphate buffered saline (PBS, pH 7.4) to remove imidazole. The empty pET32a was used for producing control histidine-tagged protein, which was expressed and purified identical to the procedure for the cystatin-histidine-tagged fusion protein. The purity of the purified rHCcyst-2 was analyzed by 12 % sodium dodecyl sulfate polyacrylamide gelelectrophoresis (SDS-PAGE) followed by Coomassie blue staining. Protein concentrations were determined by Bradford method. LPS was depleted from the rHCcyst-2 using Detoxi-Gel Affinity Pak pre-packed columns (Thermo Fisher Scientific, Waltham, MA, USA). The concentrations of the recombinant proteins were equalized to 1 mg/ml prior to LAL assay. Endotoxin levels of the protein samples were measured by LAL gel clot assay using a Pyrosate^®^Kit (Cape Cod Inc., East Falmouth, MA, USA). The samples whose endotoxin content was less than the sensitivity of the Pyrosate kit (<1EU per 1 mg of the recombinant proteins) were collected for the subsequent experiments.

### Generation of polyclonal antibodies

The goat antisera used in western blot analyses were collected from five goats experimentally infected with *H. contortus*. The goats were raised in helminths-free conditions and then orally challenged with 5000 infective L3. One month later, the goat antisera were collected and stored at −70°C until use.

To generate polyclonal antibodies against rHCcyst-2, 0.3 mg of purified rHCcyst-2 was mixed with Freund's complete adjuvant (1:1) and injected into SD rats subcutaneously in multiple places, following the method described by Wang et al. [40]. After the first injection, rats were then boosted four times at 2-week intervals with the same dose. The sera containing specific anti-rHCcyst-2 antibodies were harvested 10 days following the last injection and the specific reactivity with rHCcyst-2 was checked by enzyme-linked immunosorbent assay (ELISA).

### Western blot analysis

Purified rHCcyst-2 (20 μg) was resolved on 12 % SDS-PAGE and transferred to Hybond-C extra nitrocellulose membranes (Amersham Biosciences, UK). Non-specific binding sites were blocked by immersing the membranes in 5 % skim milk in Tris-buffered saline (TBS) for 1 h at room temperature. The membranes were then washed 5 times (5 min each) in TBS containing 0.1 % Tween-20 (TBST). Subsequently, the membranes were incubated with the primary antibodies (antiserum from goats experimentally infected with *H. contortus*) for 1 h at 37°C (dilutions 1:100 in TBST). After being washed 5 times in TBST, the membranes were then incubated with HRP-conjugated rabbit anti-goat IgG (Sigma, St. Louis, MO, USA) for 1 h at 37°C (diluted 1:2000 in TBST). Finally, the immunoreaction was visualized using freshly prepared diaminobenzidine (DAB, Sigma) as a chromogenic substrate after 5 min.

### Localization of HCcyst-2 by immunohistochemical study

Washed adult worms suspended in PBS were fixed in 4 % formaldehyde-0.2 % glutaraldehyde in PBS for 90 min and then immersed in TISSUE-TeK^®^ O.C.T. compound (SAKURA, USA). They were snap frozen in liquid nitrogen and stored at -20°C until required for further processing. Cryostat sections of 10 μm thickness were cut, washed with PBS, and treated for 60 min with 10 % normal goat serum in PBS to prevent non-specific binding of antibodies. The sections were then incubated with specific rat anti-rHCcyst-2 antiserum (1:100 dilution) or normal rat serum (control) for 60 min at 37 °C, washed 15 min × 3 with PBS, and subsequently incubated for 60 min with Cy3 goat anti-rat IgG (ab6953, Abcam, Cambridge, MA, USA). Finally, the sections were stained with DAPI (Beyotime, Haimen, Jiangsu, China) to show DNA. After washing with PBS, the specimens were immersed in Anti-Fade Fluoromount solution (Beyotime), which prevents fading of fluorescence during microscopic examination.

### Proteinase inhibition assays

To calculate the inhibitory activity of the recombinant protein, the concentration of rHCcyst-2 at which a 50 % inhibition of the proteolytic enzymes activities achieved (IC50) was measured [41]. Recombinant protein was pre-incubated with each enzyme in an assay buffer for 30 min. Then, 0.25 mM of the protease-specific substrates was added to each well and residual enzyme activity monitored. The histidine-tagged protein was used as control. Enzymes used were as follows: human cathepsin L (0.05 μM), human cathepsin B (0.05 μM), human caspase 1 (0.05 μM) as well as papain (0.15 μM). All of these enzymes were purchased from Sigma Company. The assay buffer used consisted of 100 mM sodium acetate, pH 5.5, 100 mM NaCl, 1 mM EDTA, 1 mg/ml cysteine and 0.005 % TritonX-100. The substrates purchased (Sigma company) were as follows: Z-Phe-Arg-AMC-HCl for papain, cathepsin L and cathepsin B and Ac-Tyr-Val-Ala-Asp-AFC for caspase 1. Fluorescence intensity was monitored by SPECTRAFLUOR (TECAN, Maennedorf, Switzerland) with the wave length pair of 360-460 nm for emission and excitation respectively.

### Isolation of goat monocytes

Peripheral blood mononuclear cells (PBMCs) were separated from heparinized blood with the standard Ficoll-hypaque (GE Healthcare, USA) gradient centrifugation method and washed twice in PBS. Monocytes were isolated by their adherence to plastic surface [42]. The goat PBMCs were seeded in a 6 wells flat-bottom tissue culture plates (Corning, USA) in cell culture medium RPMI 1640 (GIBCO, UK) containing 10 % heat inactivated fetal calf serum (GIBCO, UK), 100 U/mL penicillin and 100 mg/mL streptomycin (GIBCO, UK). Plates were incubated at 37 °C in a humidified atmosphere with 5 % CO2 for 1 h [43]. Non-adherent cells were removed by washing twice with PBS. The adherent cells were collected and adjusted to a density of 1×10^6^ cells/mL in cell medium at 37 °C in a humidified atmosphere with 5 % CO2. Cell viability, as determined by trypan blue dye exclusion was more than 95 % in all cases.

### Uptake the rHCcyst-2 by goat monocytes

Freshly isolated goat monocytes were seeded into 24-well plates with rHCcyst-2 (40 μg/ml). The non-treated cells were set as control. After incubation at 37 °C for 30 min, cells were collected and washed twice with ice-cold PBS. The IF analyses were performed on 4 % paraformaldehyde-fixed monocytes (rHCcyst-2 treatment and control) plated on 0.01 % poly-L-lysine-coated slides). Then, cells were permeabilized by incubation for 5 min in 0.5 % TritonX-100 in PBS, and were treated with a blocking solution (2 % BSA in PBS) for 30 min to reduce background staining. After sequential incubation with specific rat anti-rHCcyst-2 antiserum (1:100 dilution) for 2 h and incubation with Cy3 goat anti-rat IgG (ab6953, Abcam) for 1 h, DIO and DAPI (Beyotime) were used for plasma membrane and nucleus staining, respectively for 6 min each. Then, protein localization was determined by observing the staining patterns with a 100×oil objective lens on a laser scanning confocal microscope (LSM710, Zeiss, Jena, Germany). Exposure conditions were applied uniformly for each color channel. All procedures were carried out at room temperature. Digital images were captured using the Zeiss microscope software package ZEN 2012 (Zeiss).

### Detection of cytokine secretion

To determine cytokine secretion, goat monocytes were stimulated with LPS (100 ng/ml) for 72 h in the presence or absence of rHCcyst-2. The supernatants were collected and cytokine testing was performed by ELISA. The levels of TNF-α, IL-1β, IL-10, IL-12p40 and TGF-β1 in supernatants were determined using commercially available goat ELISA kits (Anoric, Tianjin, China). The analysis was performed with the data from three independent experiments.

### Analysis of MHC molecule expression

The purified monocytes (0.5×10^6^ cells/ml) were incubated with different concentrations of rHCcyst-2 or equal volumes of control buffer for 24 h in complete RPMI 1640 at 37 °C. Cells were then stained with the monoclonal antibodies to MHC-I (MCA2189A647, AbDserotec, BioRad Laboratories, CA, USA) and MHC-II (MCA2226F, AbDserotec), and analyzed on a FACS Calibur cytometer (BD Biosciences, San Jose, CA, USA). Results were expressed as the percentage of mean fluorescence intensity (MFI) of control.

### Measurement of nitric oxide production

To determine nitric oxide production, goat monocytes were stimulated with LPS (100 ng/ml) for 48 h in the presence or absence of rHCcyst-2. NO was measured in the cell supernatants as nitrite using a NO assay kit (Beyotime) according to the manufacturer's protocol. Briefly, a standard curve was prepared with standard nitrite solutions in DMEM medium. The standard solutions or cell supernatants were reacted with nitrate reductase for 30 min in a 96-well plate, and then Griess reagent I and Griess reagent II were added. After 10 min incubation at room temperature, the absorbance at 540 nm was read in a microplate reader (Bio-Rad, Hercules, CA, USA). The samples were assayed in triplicate.

### FITC-dextran internalization

To confirm the effect of rHCcyst-2 on the phagocytotic ability of goat monocytes, the FITC-dextran internalization of cells were analyzed by flowcytometry. Cells were collected after treated with rHCcyst-2 for 48 h and incubated with FITC-dextran (1 mg/ml in RPMI1640) for 1 h at 37°C. Cells added with the same amount of FITC-dextran and incubated at 4°C for 1 h were used as the baseline of monocyte phagocytosis. After incubation, cells were washed extensively to remove excess FITC-dextran. The FITC-dextran internalization of cells were analyzed by flowcytometry (BD Biosciences) using Cell QuestSoftware and median fluorescence intensity (MFI) was calculated.

### Statistical analysis

Data are expressed as mean ± the standard deviation of the mean. Statistical analysis for significant differences was performed using an analysis of variance, the Student's *t* test for parametric samples (GraphPad Prism, USA).
